# Antifibrotic effect of novel neutrophil gelatinase-associated lipocalin inhibitors in cardiac and renal disease models

**DOI:** 10.1038/s41598-021-82279-0

**Published:** 2021-01-28

**Authors:** Benjamin Bonnard, Ernesto Martínez-Martínez, Amaya Fernández-Celis, Marie Pieronne-Deperrois, Quoc-Tuan Do, Isbaal Ramos, Patrick Rossignol, Faiez Zannad, Paul Mulder, Antoine Ouvrard-Pascaud, Natalia López-Andrés, Frédéric Jaisser

**Affiliations:** 1 INSERM, UMRS 1138, Centre de Recherche des Cordeliers, Sorbonne Université, Université de Paris, 15 rue de l’Ecole de Médecine, 75006 Paris, France; 2 Cardiovascular Translational Research, Navarrabiomed (Miguel Servet Foundation), Instituto de Investigación Sanitaria de Navarra (IdiSNA), Pamplona, Spain; 3 Inserm U1096, UFR Médecine-Pharmacie, Rouen, France; 4 Greenpharma SAS, Orléans, France; 5 Innovative Technologies in Biological Systems SL (INNOPROT), Bizkaia, Spain; 6 INSERM Centre d’Investigations Cliniques-Plurithématique 1433, UMR 1116, CHRU de Nancy, French-Clinical Research Infrastructure Network (F-CRIN) INI-CRCT, Université de Lorraine, Nancy, France

**Keywords:** Drug discovery, Drug screening, Target validation

## Abstract

Neutrophil gelatinase-associated lipocalin (NGAL) is involved in cardiovascular and renal diseases. Gene inactivation of NGAL blunts the pathophysiological consequences of cardiovascular and renal damage. We aimed to design chemical NGAL inhibitors and investigate its effects in experimental models of myocardial infarction (MI) and chronic kidney disease induced by 5/6 nephrectomy (CKD) on respectively 8–12 weeks old C57Bl6/j and FVB/N male mice. Among the 32 NGAL inhibitors tested, GPZ614741 and GPZ058225 fully blocked NGAL-induced inflammatory and profibrotic markers in human cardiac fibroblasts and primary mouse kidney fibroblasts. The administration of GPZ614741 (100 mg/kg/day) for three months, was able to improve cardiac function in MI mice and reduced myocardial fibrosis and inflammation. The administration of GPZ614741 (100 mg/kg/day) for two months resulting to no renal function improvement but prevented the increase in blood pressure, renal tubulointerstitial fibrosis and profibrotic marker expression in CKD mice. In conclusion, we have identified new compounds with potent inhibitory activity on NGAL-profibrotic and pro-inflammatory effects. GPZ614741 prevented interstitial fibrosis and dysfunction associated with MI, as well as tubulointerstitial fibrosis in a CKD model. These inhibitors could be used for other diseases that involve NGAL, such as cancer or metabolic diseases, creating new therapeutic options.

## Introduction

Neutrophil gelatinase-associated lipocalin (NGAL) (also known as lipocalin-2, oncogene 24p3, siderocalin, or uterocalin) is a small circulating protein induced in a wide variety of pathological situations. Initially, NGAL was identified in mature neutrophil granules and shown to also be expressed in the kidney, prostate, and various epithelia^1^. Over the last decade, NGAL has been shown to have a broad expression pattern and is expressed by renal, endothelial, and smooth muscle cells and hepatocytes, as well as cardiomyocytes, neurons, and various populations of immune cells, such as macrophages and dendritic cells.

Several studies have highlighted NGAL as a potent early biomarker of renal injury or as an inflammation biomarker^2,3^. Other studies have shown that NGAL has a role in iron trafficking and chemotactic and bacteriostatic effects, as well as in differentiation, proliferation, and inflammation^4–7^. NGAL participates in the epithelial-mesenchymal transition in vivo, as shown in a pulmonary adenocarcinoma model^8^, and in vitro in prostate and breast-cancer cells^9^. In these models, NGAL promotes the motility, invasiveness, and metastatic capacities of cancer cells. More recently, NGAL has also been shown to be involved in cardiovascular, metabolic, and renal diseases^10^. Indeed, gene inactivation in mice blunts the pathophysiological consequences of cardiovascular (myocardial infarction or ischemia)^11,12^, renal (subtotal nephrectomy)^13^, and metabolic (high-fat diet)^14^ challenges.

Overall, these data suggest that NGAL is a potential therapeutic target in several diseases. There is thus a need to find NGAL inhibitors that can be used as therapeutic agents in various disease settings. More precisely, we aimed to design chemical NGAL inhibitors and investigate the protective effects of pharmacological NGAL blockade in cardiac and renal fibrosis. We thus performed in vitro studies in human cardiac fibroblasts and primary mouse renal fibroblasts and in vivo experiments in myocardial infarction (MI) and chronic kidney disease (CKD) mouse models.

## Results

### Selection of potential disruptors of the NGAL-NGAL receptor interaction

We prepared a database of compounds for virtual screening and built in silico screening models for NGAL-interacting compounds prior to the virtual screening of compounds on NGAL-modulated pathways.

We used the www.ambinter.com database, containing more than 35 million molecular references. Our aim was to have access to as many molecules as possible for future virtual and real screening. These chemical catalogues are generally found in various formats that are not directly exploitable. We developed a procedure to “normalize” the structures that would go into the database: discard radioactive, metallic, products, or reactive products, pan-assay interfering compounds, separate salts from the main compounds, normalize the informatic representation of the chemical structures (aromatization, ionic states, etc.), and calculate the 3D structure of each compound. Natural compounds can present specific problems, such as fused rings or missing chirality information. In such cases, the most probable configurations were calculated according to their internal energies. Unity molecular fingerprints (which can be considered as bar-codes that identify molecules) were calculated for the molecules for subsequent diversity or similarity searches. Subsets of the database consisting of natural products, drug-like molecules, lead-like molecules, and molecules that interfere with protein–protein interactions were also built. A workflow for the preparation of the molecules was therefore applied for virtual screening^15^.

**Figure 1:**
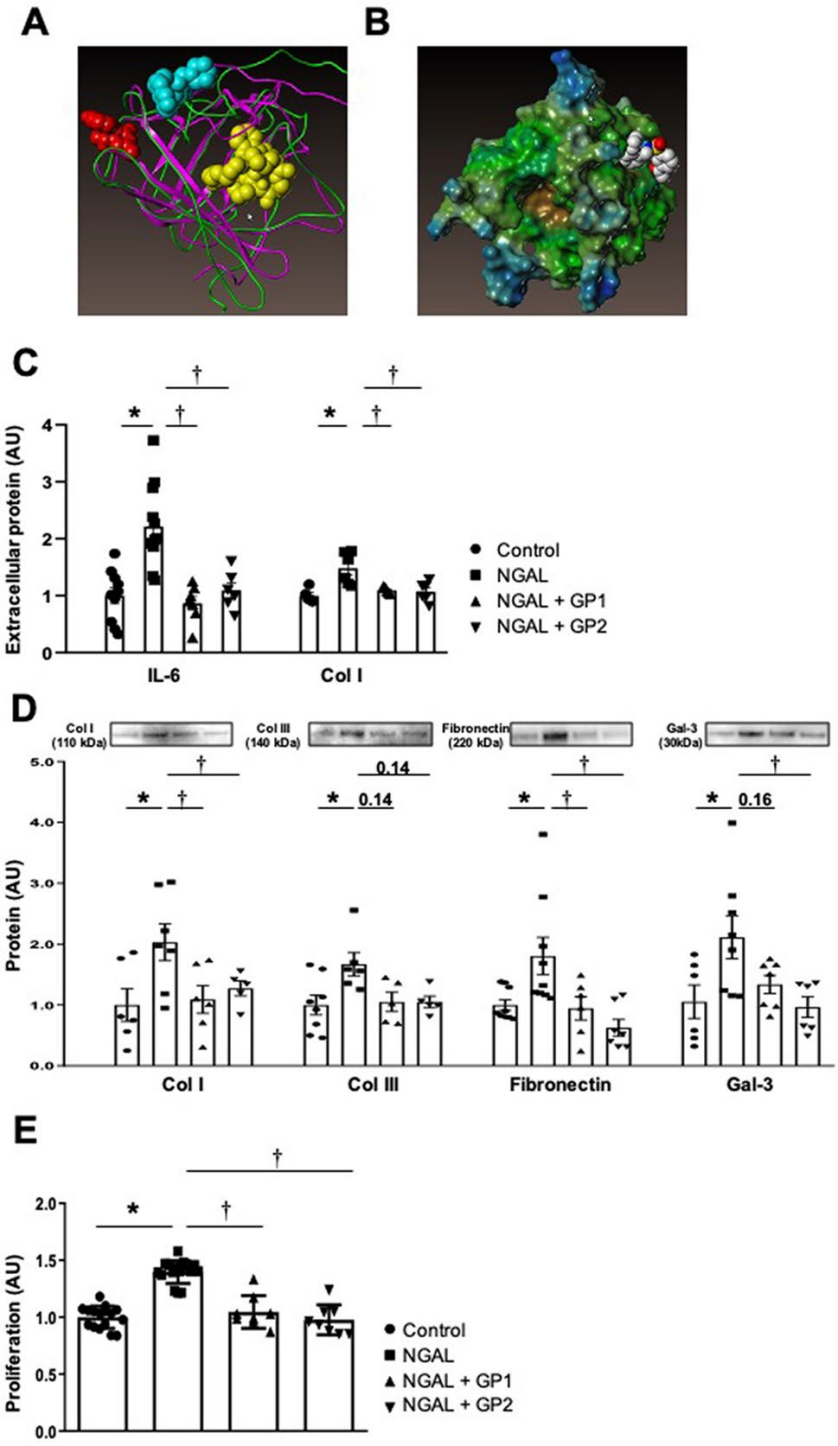
(**A**) Difference in the 3D structure of NGAL as determined by X-rays (1DVF, magenta) and NMR (1NGL, green). The main site is represented by yellow spheres and the two peripheral sites by red and cyan spheres. (**B**) Interaction of GPZ614741 with the NGAL PPI site. Effects of GP1 (GPZ614741) and GP2 (GPZ058225), at 10 µM, on (**C**) IL6 and Col I secretion, (**D**) cellular content of Col I, Col III, fibronectin, and Gal-3, and (**E**) cell proliferation in hNGAL-treated human cardiac fibroblasts. The full-length gel and bands are included in the Supplementary Fig. 10. *p < 0.05 vs. Control. ^†^p < 0.05 vs. NGAL-treated human cardiac fibroblasts.

Several crystal structures of NGAL with siderophores are published in the Protein Data Bank (https://www.rcsb.org/). Examination of these 3D structures, with or without siderophores, clearly shows that the general 3D structure of the protein does not change. Therefore, targeting the “main” binding site of siderophores may not exert significant effects, although we cannot rule this out. The biological activity of NGAL may be related to its interactions with a receptor. As the NGAL receptor could not be modelled (no crystallographic data and no reliable homologue with a known 3D structure), our strategy was based on blocking the interaction of NGAL with its receptor by identifying putative protein–protein interaction (PPI) disruptors. Based on the 3D structure of NGAL (PDB accession numbers: 1DFV & 1NGL), we surveyed potential “hot spots” (i.e. important residues for PPI) on the protein surface using ANCHOR^16^ (http://structure.pitt.edu/anchor/), an online software to analyse PPIs. We then identified two peripheral sites that may be druggable PPI zones (see Fig. 1A, red and cyan sites).

We selected potential disruptors of the NGAL-NGAL receptor interaction by virtual screening. This consisted of simulating the binding energy of a small molecule with NGAL binding sites. As there are significant differences between NMR and X-ray 3D structures of NGAL (RMSD = 4.6 Å calculated according to the backbone of 1DFV and 1NGL, see Fig. 1A), it was necessary to use these two structures as templates for modelling NGAL. Surflex-dock 2.7^17^, as implemented in Sybyl-X 2.1.1, was used for docking calculations. We identified two peripheral sites that may be druggable PPI zones. The “-pscreen” parameter was selected for virtual screening. Based on potential protein–protein interaction with NGAL, 32 potential hits were selected based on their docking score and after visual inspection. As an example, Fig. 1B shows the docking of GPZ614741 (one of the active molecules, see below) on a PPI site of NGAL.

### Inhibitory activity of compounds selected after virtual screening

We previously reported that recombinant human NGAL (hNGAL) induced expression of inflammatory (IL6) and profibrotic (Col I, Col III) markers in HCFs^11^. Thus, the inhibitory activity of the compounds was initially evaluated on hNGAL-induced IL6 secretion. Among the 32 compounds obtained by virtual screening, four compounds (GPZ614741, GPZ058225, GPZ425915 and GPZ413473) showed an important inhibitor effect on hNGAL-induced IL6 secretion (Table S1) and were tested for their effects on different intracellular fibrotic markers such as collagen I and III, fibronectin and galectin-3 (Table S2). In all cases, GPZ614741 (also named GP1) and GPZ058225 (also named GP2) were the most effectives and were selected to create a second-generation compounds derived from GPZ614741 or GPZ058225 (Table S3). The inhibitory activity of 23s-generation compounds derived from GPZ614741 or GPZ058225 were next evaluated on hNGAL-induced IL6 secretion in HCFs (Table S4). Eleven were as efficient as GPZ614741 and GPZ058225 at inhibiting hNGAL-induced IL6 secretion, whereas the other tested compounds were unable to conserve their inhibitory effect (Table S4).

GP1 and GP2 fully inhibited NGAL-induced IL6 and Col1secretion (Fig. 1C). The two selected compounds also blocked hNGAL-induced expression of intracellular profibrotic proteins (Col I, Col III, fibronectin, Gal3) (Fig. 1D), as well as cell proliferation (Fig. 1E).

To evaluate the dose dependent effect of NGAL inhibitor, we performed a dose–response of GP1 and GP2 on proliferation induced by NGAL in human cardiac fibroblasts (Fig. S2). Both compounds were able to block the proliferative effects of NGAL at 1 and 10 µM, but not at lower concentrations (Fig. S2). GP1 and GP2 at 1 µM did not prevent the increase in IL6 secretion induced by NGAL (Fig. S3), indicating that 10 µM is an efficient concentration to inhibit IL6 secretion induced by NGAL in HCF cells. In mouse kidney fibroblasts (MKFs) co-treated with NGAL + GP1 at 1 µM, collagen I expression was prevented but not those of fibronectin, αSMA, IL6 and MCP1 (Fig. S4). To assess the specificity of GP1 for NGAL, we treated HCFs and MKFs with recombinant TGFβ, a known profibrotic and proinflammatory stimulus. TGFβ increased IL6 and Col I secretion in HCFs (Fig. S5) and induced gene expression of Col I, Fibronectin and IL6 in MKFs (Fig. S6). GP1 has no inhibitory effect over the TGFβ effects, supporting the view that GP1 has no off-target effects on profibrotic and proinflammatory pathways (Figs. S5,S6).

### Cell toxicity and absorption, distribution, metabolism, and excretion (ADME) tests

An ADME-Tox panel (Table S5) showed GP1 to be nontoxic for hepatocytes (MTT, up to 0.1 mM) and cardiomyocytes (hERG predictor assay). The PPB was low and fully stable to acidic pH. It was also perfectly soluble in aqueous buffers and partially processed by CYP3A4. The ADME-Tox panel showed GP2 to have no significant hepatotoxicity (> 0.1 mM) or cardiotoxicity (it did not block the hERG channel). GP2 bound to plasma proteins within an acceptance range. It showed mild solubility problems (only 63% in the solubility test) and the CYP3A4 isoform appeared to be involved in its processing (inhibited the enzyme activity in a competition assay by 32%) (Table S5).

Based on the efficacy and ADME-TOX results, we next tested whether oral administration of GP1 (GPZ614741) could efficiently prevent the cardiac and renal impact of MI or subtotal nephrectomy in vivo, respectively.

### Impact of GP1 (GPZ614741) in a myocardial infarction mouse model

We previously reported that *Lcn2* (NGAL) gene inactivation blunts the functional and morphological consequences of MI^11^. We therefore tested whether the GP1 compound mimicked the genetic inactivation of *Lcn2*/NGAL in a murine MI model. As shown in Table 1, three months administration of GP1 (100 mg/kg/day in the food) induced a significant increase in fractional shortening (FS) resulting from a slight, statistically non-significant decrease in both the LV end diastolic and systolic diameters (LVDD and LVSD, respectively) relative to those of the non-treated MI group. At the same time-point, both stroke volume (SV) and cardiac output (CO) were higher in the GP1-treated group than the non-treated MI group.

**Table 1:** Cardiac parameters in the myocardial infarction animal model and renal parameters in the chronic kidney disease animal model.

Cardiac parameters	Sham	MI	MI + GP1
HW/TL (mg/mm)	8.5 ± 0.4	10.9 ± 0.4*	11.3 ± 0.8*
LVW/TL (mg/mm)	6.2 ± 0.3	8.3 ± 0.3*	8.8 ± 0.6*
LVEDD (mm)	3.33 ± 0.08	6.31 ± 0.12*	5.88 ± 0.39*
LVESD (mm)	2.03 ± 0.05	5.47 ± 0.13*	4.90 ± 0.39*
FS (%)	39 ± 0.93	13.4 ± 1.0*	17.2 ± 1.3*
SV (ml/beat)	0.092 ± 0.003	0.062 ± 0.005*	0.086 ± 0.004^†^
CO (ml/min)	39.7 ± 2.0	29.4 ± 1.9*	47.1 ± 3.3^†^
LVEDP (mmHg)	2.73 ± 0.95	5.91 ± 0.49*	4.41 ± 0.57
LVESP (mmHg)	85.05 ± 1.39	78.82 ± 2.76	82.31 ± 4.22
LV (dP/dt_max_)	5220 ± 229	3894 ± 259*	4501 ± 320
LV (dP/dt_min_)	4874 ± 550	3117 ± 253*	4487 ± 413^†^
tau e	6.05 ± 0.30	7.97 ± 0.45*	6.08 ± 0.27^†^
tau 1/2	4.43 ± 0.20	5.82 ± 0.32*	4.33 ± 0.21^†^

Renal parameters	Sham	CKD	CKD + GP1
KW/TL (mg/mm)	9.40 ± 0.26	11.5 ± 0.42*	11.3 ± 0.23*
SBP (mmHg)	115 ± 0.75	139.3 ± 2.22*	122.1 ± 2.58^†^
Plasma creatinine level (mM)	4.14 ± 1.30	21.5 ± 1.45*	20.4 ± 1.54*
Plasma urea level (mM)	7.11 ± 0.34	15.2 ± 0.36*	16.74 ± 1.86*

*HW/TL* heart weight/tibia length; *LVW/TL* left ventricle weight/tibia length; *LVEDD* left ventricular end diastolic diameter; *LVESD* left ventricular end systolic diameter; *FS* Fractional shortening; *SV* stroke volume; *CO* cardiac output; *LVEDP* left ventricular end systolic pressure; *LVESP* Left ventricular end systolic pressure; *LV dP/dT*_*max*_* and dP/dT*_*min*_ contractility and relaxation index, respectively; *tau-e and tau ½* relaxation constants; *KW/TL* kidney weight/tibia length; *SBP* systolic blood pressure.

*p < 0.05 vs*.* Sham.

^†^p < 0.05 vs. MI or CKD mice.

Three months of treatment with GP1 (100 mg/kg/day in the food) did not modify the LV end-systolic pressure (LVESP) relative to that of the MI non-treated group and resulted in a trend towards an increase in the LV dP/dt_max_ (p = 0.07) (Table 1). Moreover, the LV end-diastolic pressure (LVEDP) tended to be lower in the MI GP1-treated group, whereas the increase in the LV dP/dt_min_, together with the decrease in the LV relaxation constant Tau, indicated an improvement in diastolic relaxation upon chronic treatment.

**Figure 2:**
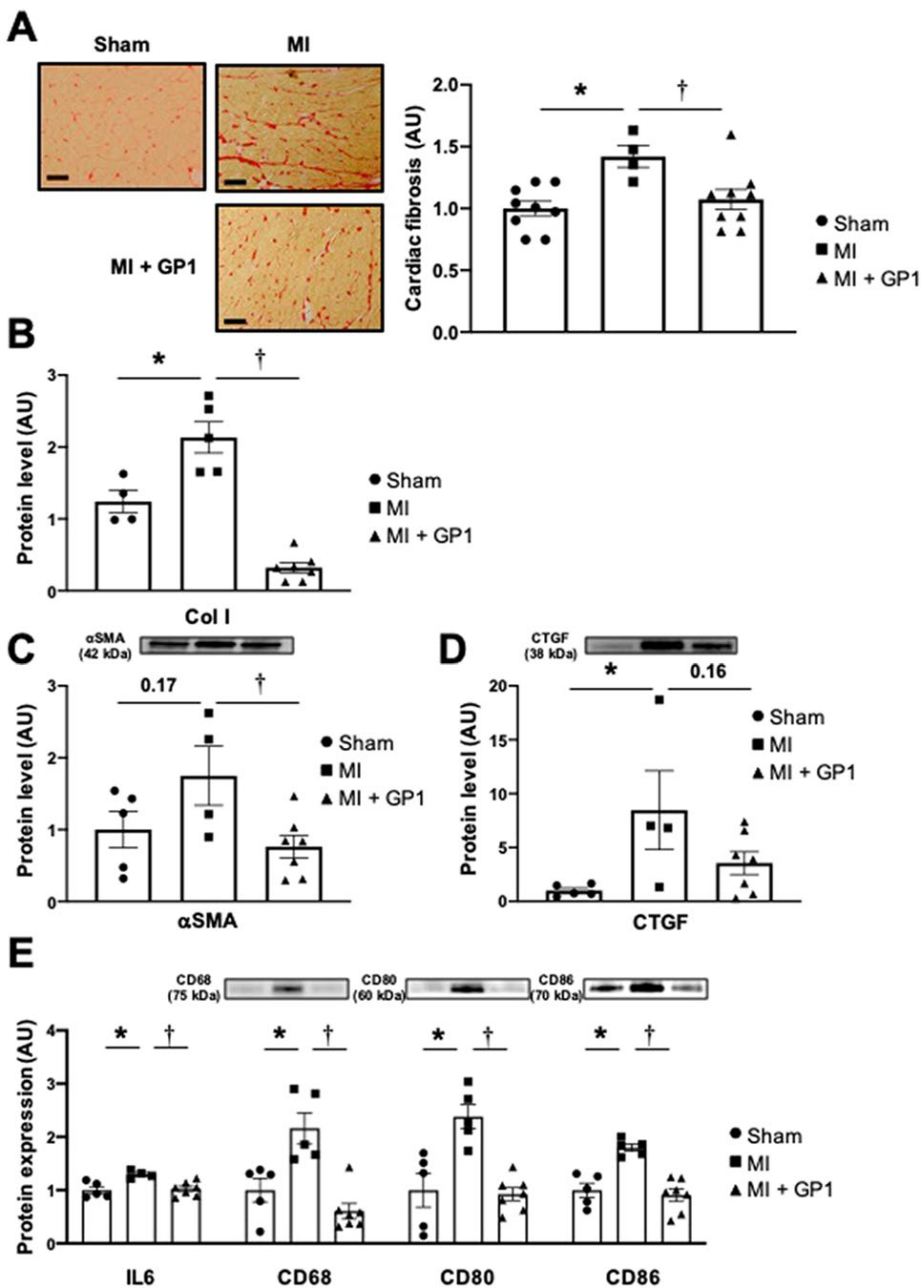
Effect of three months of GP1 (GPZ614741) administration. (**A**) Representative microphotographs and quantification of interstitial fibrosis. (**B**) Col I, (**C**) αSMA, (**D**) CTGF and (**E**) proinflammatory markers (IL6, CD68, CD80, and CD86) protein levels in Sham, MI, and MI + GP1 (GPZ614741)-treated mice. The full-length gel and bands are included in the Supplementary Fig. 11. *p < 0.05 vs*.* Sham. ^†^p < 0.05 vs. MI mice.

Treatment with GP1 (100 mg/kg/day in the food) resulted in a significantly lower LV interstitial collagen deposition than in the non-treated MI group (Fig. 2A). Three months of treatment with GP1 significantly prevented the upregulation of Col I, αSMA and CTGF observed in the non-treated MI group (Fig. 2B–D). The infarct size in mid LV sections was similar between GP1-treated vs. untreated infarcted mice (infarct size %: untreated 39.0 ± 2.3, GP1-treated 40.0 ± 1.8, n = 4–9, NS). Permanent coronary artery ligation induces transmural infarction and the inhibition of NGAL did not affect collagen levels in the infarct zone which contained only fibrosis (Fig. S7). Treatment with GP1 significantly prevented the upregulation of cardiac protein levels of IL6 and the expression of inflammatory markers, such as CD68 (marker of monocytes lineage), CD80, and CD86 (markers of macrophages) (Fig. 2E).

### Impact of GP1 (GPZ614741) on renal fibroblasts and the kidney in a CKD mouse model

We next tested whether GP1 blunted recombinant mNGAL-induced expression of profibrotic/proinflammatory markers in mouse primary renal fibroblasts (MKF) and whether in vivo administration of GP1 in the 5/6 Nx CKD mouse model had a similar effect as that reported for *Lcn2*/NGAL gene inactivation^13^.

**Figure 3:**
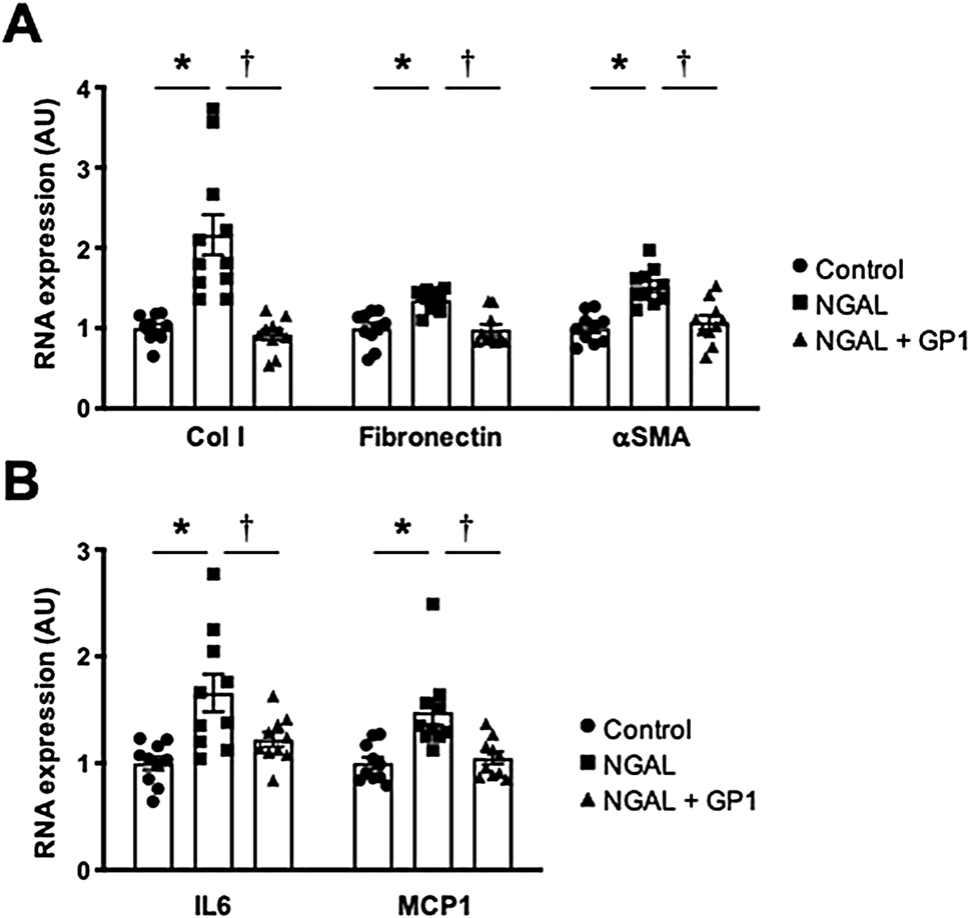
Effect of GP1 (GPZ614741), at 10 µM, on mRNA levels of (**A**) profibrotic markers Col I, fibronectin and αSMA) and (**B**) proinflammatory markers (IL6 and MCP1) in mNGAL-treated MKF cells. *p < 0.05 vs*.* Control. ^†^p < 0.05 vs. NGAL-treated MKF cells.

MKF were stimulated with recombinant murine NGAL (mNGAL), with or without GP1. GP1 inhibited mNGAL-induced expression of profibrotic (Col I, fibronectin, αSMA) (Fig. 3A) and inflammatory (IL6, MCP1) markers (Fig. 3B).

In vivo administration of GP1 had no impact on functional parameters, such as plasma levels of urea or creatinine (neither one nor two months after CKD induction) (Table 1), indicating that GP1 did not blunt renal dysfunction associated with CKD in the 5/6 nephrectomy model. However, two months of GP1 administration prevented the increase in blood pressure observed in this CKD model (Table 1).

**Figure 4:**
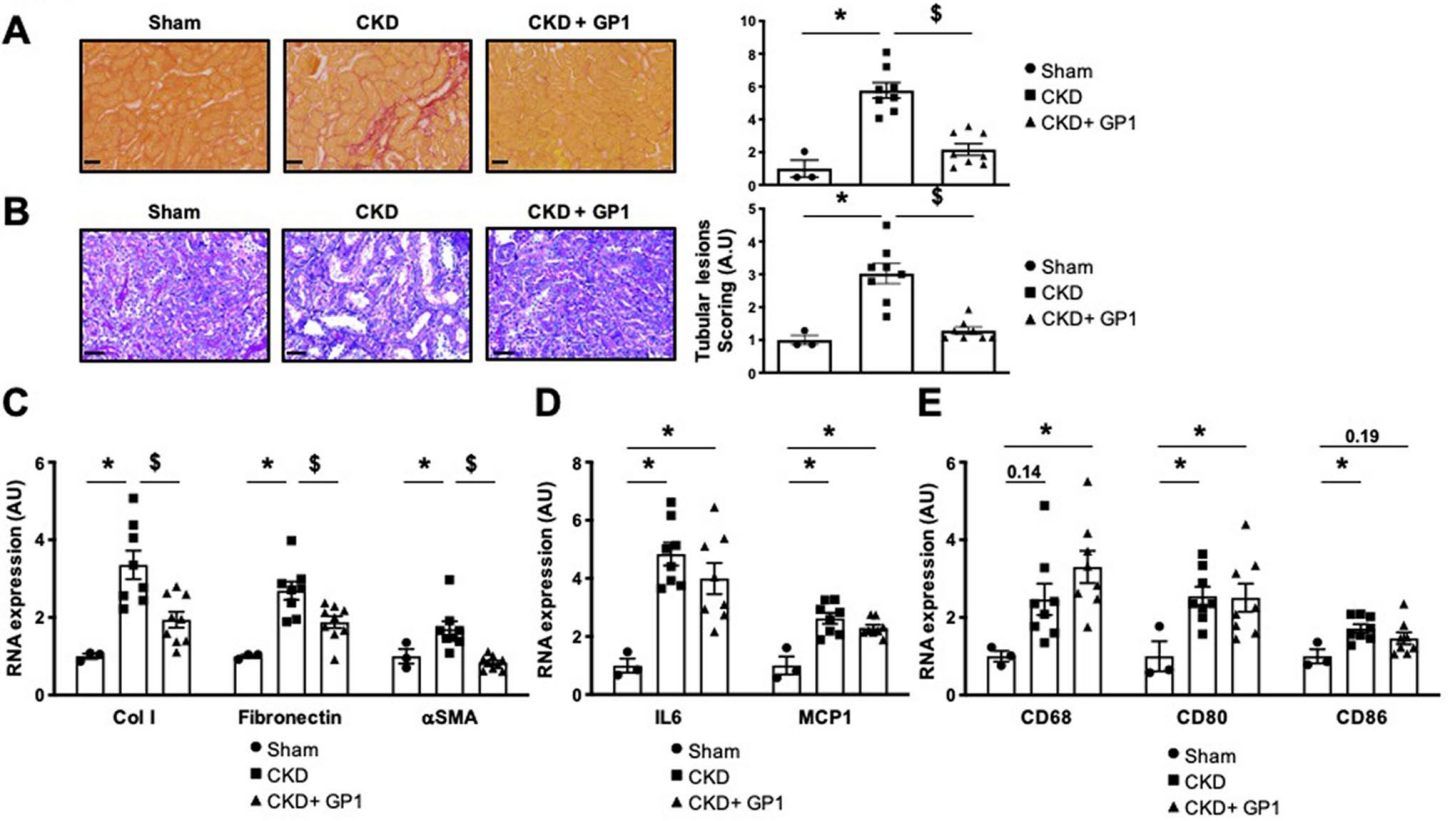
Effect of two months of GP1 (GPZ614741) administration. (**A**) Representative microphotographs and quantification of Sirius red staining and (**B**) Periodic acid-Schiff staining (scale bar 50 μm), and (**C**) mRNA expression of profibrotic markers (Col I, fibronectin and αSMA), (**D**) proinflammatory markers (IL6 and MCP1), and (**E**) CD68, CD80, and CD86 in a CKD mouse model. *p < 0.05 vs*.* Sham. ^†^p < 0.05 vs. CKD mice.

We next analyzed whether the anti-fibrotic and anti-inflammatory effects observed in the MI model were also present in the mouse CKD model. Two months of GP1 administration had a strong antifibrotic effect in vivo by blunting the renal tubulointerstitial fibrosis associated with CKD (Fig. 4A). Both CKD and CKD + GP1 mice did not show collagen deposition in glomeruli compared to Sham group (Fig. S8). Tubular lesion scoring revealed that GP1 prevented tubular injury induced by CKD (× 3.1 in CKD vs. Sham mice) (Fig. 4B). GP1 administration also blunted the increased expression of profibrotic markers (Col I, fibronectin, αSMA) (Fig. 4C) but not those of inflammatory markers, such as IL6 and MCP1 (Fig. 4D), CD 68, CD80, or CD86 (Fig. 4E).

## Discussion

We have identified several compounds with potent inhibitory activity on the NGAL-mediated expression of profibrotic and pro-inflammatory markers and cell proliferation in two primary cell cultures of fibroblasts of human cardiac or murine renal origin. One of the compounds, GP1, showed efficient in vivo activity to prevent the interstitial fibrosis associated with MI and CKD. The effects of GP1 (GPZ614741) on cardiac function, fibrosis, and inflammation mimics the functional, molecular, and histological impact of global genetic inactivation of *Lcn2* in the same MI model that we previously reported in Martinez-Martinez et al.^11^. In the CKD model, the effect was only partial relative to global genetic *Lcn2* inactivation. Although the effect of GP1 on renal fibrosis mimicked the effect of global genetic inactivation of *Lcn2,* it did not improve renal function or inflammation. The reason for these differences may be related to heterogenous intrarenal tissue distribution of the compound, which may thus, for example, not have an effect on glomerular function. The absence of effect on renal inflammatory markers is more puzzling, given the good inhibitory effect of GP1 on NGAL-induced inflammatory gene expression in primary kidney fibroblasts, as well as in vivo in the MI model. Of note, GP1 blunted the increased blood pressure normally associated with CKD. This may be related to the vascular impact of NGAL, which has indeed been previously reported upon metabolic challenge in mice^14^. Further studies are required to determine the possible mechanisms related to blood pressure control.

NGAL/Lipocalin-2 (LCN-2) is a member of the broader lipocalin family, which includes over 20 soluble proteins, most of which are extracellular^1^. This family is evolutionarily conserved and found in various lineages from bacteria to plants, invertebrates, and vertebrates^18^. Lipocalins have a three-dimensional structure (lipocalin domain) in which eight β-strands form a symmetrical β-barrel fold with a cylindrical shape. The β-barrel structure provides a hydrophobic cavity to bind to a variety of lipophilic ligands, including retinoic acid, progesterone, prostaglandin, fatty acids, steroids, leukotriene B4, and platelet activating factor^1,18^. NGAL/Lipocalin 2 is produced by a variety of cells of the cardiovascular and renal systems, adipocytes, and inflammatory cells and is also found in brain, bone, gut, liver, etc. Two different cell-surface receptors for NGAL/LCN2 have been described: solute carrier family 22 member 17 (SLC22A17, 24p3R or NGALR2) and low-density lipoprotein-related protein 2 (LPRP2 or megalin). High levels of 24p3R are expressed by specific cells, including macrophages, neutrophils, lung and gut epithelia, kidney epithelial cells, vascular cells and cardiomyocytes, astrocytes, microglia, and neurons. Megalin, a multi-ligand endocytic receptor, is highly expressed by certain types of absorptive epithelial cells, such as thyroid cells, epididymal cells, renal proximal tubule cells, and neuro-epithelium. Sensory organs, including the eye (specifically, retinal ganglion cells) and ear, also express megalin^18^.

Such a broad expression pattern of NGAL/LCN2 and its receptors suggests a potential role in a multitude of physiological and/or pathological processes. The use of mouse models with genetic inactivation of *Lcn2* has allowed exploration of these roles, revealing both detrimental and beneficial effects of NGAL/LCN2. Global *Lcn2* gene inactivation has beneficial effects in the cardiovascular system (recently summarized in Buonafine et al.)^19^. However, contrasting results have been reported in the kidney, for which the injection of recombinant NGAL in acute kidney injury provides a benefit^20^ but NGAL/LCN2 has a deleterious role in CKD progression^13,21^. As reported here, NGAL/LCN2 plays a major role in end-organ damage and organ fibrosis in both cardiac- and renal-disease models. *Lcn2* knockout mice also revealed the important metabolic effects of NGAL/LCN2, with higher body and adipose tissue weight in *Lcn2* KO than wild type mice, as well as insulin resistance, but a benefit of LCN2 was also been reported in beiging of adipocytes and increased thermogenesis^22^. Detrimental roles of NGAL/LCN2 have been shown in cancer. NGAL/LCN2 induces proliferation and stimulates AKT and HIF1-α activation, thus promoting angiogenesis and invasion. Moreover, NGAL can form a complex with MMP9, increasing ECM degradation and favoring metastasis^23^. Finally, NGAL/LCN2 is a pleiotropic mediator of various inflammatory processes. Lipocalin-2 has been shown to cause M1 macrophage polarization, while suppressing the formation of the M2 macrophage phenotype, to favor inflammation via the recruitment of inflammatory cells, such as neutrophils, and the induction of pro-inflammatory cytokines.

On the other hand, NGAL/LCN2 is required to limit infection. LCN2 was indeed first identified as a neutrophil granule component with a strong binding affinity to bacterial siderophores. As iron is essential for bacterial growth, NGAL/LCN2 has a bactericidal affect against a number of bacterial species by chelating siderophore-bound iron and making it unavailable for bacterial use. NGAL/LCN2 also plays an important role in immune activation during acute infection. However, LCN2 was shown to worsen the outcome in pneumococcal pneumonia infection by deactivating alveolar macrophages in an animal model^24^. NGAL/LCN2 also acts as a source of iron to *Mycobacterium tuberculosis* in infected macrophages, facilitating mycobacterial growth in vivo.

Overall, NGAL/LCN2 plays a critical role in the differential regulation of inflammation during acute infections and the progression of several chronic diseases. This Janus face/dual role suggests that NGAL/LCN2 could be an important therapeutic target in chronic diseases, but caution must be exercised in the acute setting, especially during infectious diseases in which limiting the action of NGAL/LCN2 may be deleterious.

Several ways could be used to modulate NGAL/LCN2 expression and/or activity. A few studies have used anti-NGAL antibodies, demonstrating neutralizing activity in vivo in the mouse. Anti-NGAL antibody treatment attenuated immune-cell recruitment, such as neutrophil and macrophages, in a cardiac ischemia reperfusion (IR) injury model, along with suppressed M1 marker expression and increased M2 marker expression^25^. The role of NGAL in neutrophil recruitment has also been assessed in the liver; neutralization of NGAL by antibody injections protected mice from hepatic injury and neutrophil infiltration induced by alcohol challenge^26^. Similarly, neutralizing NGAL antibodies reduced Ly6G neutrophil infiltration in two different psoriasis models^27^, leading to the diminution of proinflammatory gene expression (IL6, IL8, IL17, IL1β and IL23)^6^. In patients with an abdominal aortic aneurysm (AAA), NGAL levels are elevated in the plasma and AAA tissue^28,29^. In a mouse model of AAA, Tarín et al. showed that genetic deletion of *Lcn2* or neutralization of NGAL with polyclonal anti-NGAL antibody injections protected against AAA-induced lesions, with lower neutrophil infiltration and the diminution of MMP activity^30^. In contrast, Saha et al. showed that antibody-mediated neutralization of NGAL exacerbates DSS-induced colitis^31^, highlighting a protective role of NGAL in this model. Anti-NGAL antibodies lower tissue iron concentrations in a dose-dependent manner, which could starve cancer cells, thus reducing metastasis^32^. In cancer, NGAL is known to promote EMT through the alteration of E/N-cadherin expression^9^. Treatment of MCF-7 breast cancer cells (which have a metastatic tumor cell phenotype) with anti-NGAL antibodies prevented changes in E/N-cadherin expression relative to their control^33^ and anti-NGAL antibody administration decreased lung metastasis in a mouse model of breast cancer^23^.

Another possible therapeutic approach would be to reduce NGAL expression by RNA interference. Guo et al. engineered an encapsulating liposome delivery system to target LCN2 siRNA in cells via the intercellular adhesion molecule (ICAM)-1. This approach was used to treat breast-cancer cells, reducing angiogenesis and tumor progression in vivo in a xenograph mouse model^34^.

Our approach was to identify small molecules that interfere with NGAL signaling in human fibroblasts. We identified several candidates and provide a proof of concept study that at least one of them efficiently reduces ECM remodeling in vivo in two preclinical models of CV and renal diseases. These inhibitors could be tested in other diseases that involve NGAL, such as metabolic and cardiovascular diseases or cancer, such as breast or prostate cancers, for which genetic Lcn2 inactivation and NGAL-neutralizing antibodies have proven to be beneficial.

## Limitations

Our proof of concept study had several limitations. Full characterization of the hits we identified is now required to precisely identify binding modes and in vivo pharmacodynamic and pharmacokinetic properties in various disease models. Moreover, additional structure activity relationship studies should be performed to identify derivatives with higher efficacy. As a proof of principle, we tested in vivo only one of the NGAL inhibitors at one fixed dose. Several studies should now be performed to test various doses, as well as the other compounds that we have shown to be active ex vivo using various readouts.

## Methods

The study was carried out in compliance with the ARRIVE guidelines.

### Compounds

3-Acetyl-N-[2-(1H-pyrazol-1-yl)phenyl]methyl]-benzenesulfonamide (GPZ614741, CAS 1241512-52-6) has been synthesized according to the scheme presented in Fig. S1. To a solution of [2-(1*H*-pyrazol-1-yl)phenyl]methylamine (3.0 g) in pyridine (20 mL) was slowly added 3-acetylbenzylsulfonyl chloride (4.17 g) at 0 °C, and the mixture was stirred overnight at 115 °C. After cooling down, the reaction solution was concentrated under reduced pressure. The residue was then dissolved in dichloromethane, washed with HCl 2 N, a saturated aqueous solution of NaHCO3 and saturated brine, then dried over magnesium sulfate, and concentrated under reduced pressure. The residue was finally purified by flash column chromatography (1: 1EtOAc/petroleum ether) to give the title compound as a white solid (5.72 g, 93% yield). 1H NMR (400 MHz, CDCl3) δ 8.27 (t, J = 1.6 Hz, 1H), 8.01 (dt, J = 1.6 and 8.0 Hz, 1H), 7.96 (dt, J = 1.6 and 8.0 Hz, 1H), 7.69 (d, J = 1.2 Hz, 1H), 7.63 (d, J = 2.4 Hz, 1H), 7.48 (t, J = 8.0 Hz, 1H), 7.30–7.26 (m, 2H), 7.18–7.14 (m, 2H), 6.45 (t, J = 2.2 Hz, 1H), 4.08 (d, J = 6.4 Hz, 2H), 2.55 (s, 3H).

Other compounds used in the present study are commercialized by Ambinter (see Tables S6,S7).

### Cell toxicity and absorption, distribution, metabolism, and excretion (ADME) tests

Several parameters have been evaluated to determine ADME-Tox profile of the compounds. Two specific toxicity assays have been performed to assess hepatoxicity and cardiotoxicity. Hepatotoxicity was evaluated in isolated hepatocytes from swiss mice and determined by the thiazolyl blue tetrazolium bromide colorimetric assay (sigma, St Louis, MO, USA). Cardiotoxicity was evaluated using the commercially available Predictor hERG fluorescence polarization kit (Thermo Fisher Scientific, Illinois, USA). Several absorption, distribution, metabolism, and excretion (ADME) assays have been carried out. The following experiments were undertaken: stability in human microsomes, binding to human plasma protein, and CYP3A4 inhibition assay (Vivid CYP3A4 assay, Thermo Fisher Scientific, Illinois, USA). Physico-chemical properties were also assessed such as aqueous solubility and chemical stability.

### Cell culture

Primary human cardiac fibroblasts (HCF) were obtained from Promocell (Heidelberg, Germany) and maintained in Fibroblast Media 3 following manufacturer’s instructions (Promocell). Cells were used between passages 5 and 7. Cells were stimulated with recombinant hNGAL (500 ng/mL, R&D Systems) or with TGFβ (10 ng/mL, R&D Systems) for 24 h. To assess cell proliferation the MTT test was used following provider’s instructions (Roche). For protein analysis. Primary Mouse Kidney Fibroblasts (MKF) were isolated from Wild Type (WT) mice. Briefly, 8 weeks old mice were sacrificed by cervical dislocation and kidneys were cleaned and rinsed in cold DPBS (Dulbecco's Phosphate-Buffered Saline). The renal cortex was minced and incubated in Dulbecco’s modified eagle medium/Nutrient mixture F-12 (DMEM/F12, Sigma) containing 1 mg/mL collagenase A (Roche) during 25 min at 37 °C. The digestion was inactivated by adding culture medium (DMEM/F12 + 10% FBS) then the cell suspension was passed through a 100-μm cell strainer. After centrifugation, the pellet (containing MKF) was diluted with culture medium then plated in 75 cm^2^ culture flask. After 24 h MKF were washed with DPBS before replacement of fresh culture medium. Thereafter, culture medium was changed every 48 h. Once 70–80% confluent, MKF were trypsinized and plated in 12 or 6-well plates. For experimental use, MKF were starved with culture medium containing only 3% FBS. MKF were treated with mNGAL (500 ng/mL, R&D Systems) or with TGFβ (10 ng/mL, R&D Systems) for 6 h for gene expression analysis.

### Animal experiments

Animals were housed in a climate-controlled facility with a 12-h/12-h light/dark cycle and provided free access to food and water. Experiments were approved by the Darwin ethics committee of Sorbonne University, and conducted according to the INSERM animal care and use committee guidelines. The NGAL inhibitor GP1 was incorporated in regular chow (A04, Safe, Augy, France) at the dose of 625 mg GP1/kg of chow to achieve a low dose of 100 mg/kg/day of GP1 administered to the mice (based on a daily consumption of 4 g of chow).

Myocardial infarction (MI) was induced as previously described^11^. Briefly, Chronic Heart Failure (CHF) resulted from left ventricle MI that was induced by left coronary artery ligation in 8 week-old male mice. Mice were anesthetized with intraperitoneal injections of ketamine (90 mg/kg, Merial, France) and xylazine (3.6 mg/kg, Bayer, France), followed by artificial ventilation. Anesthesia and sedation were controlled by monitoring heart rate and by performing paw pinch reflex and corneal reflex tests, before a thoracotomy was performed. The left main coronary artery was ligated close to its origin. After surgery, analgesia was prolonged during the awakening phase by an intramuscular injection of Beprucare (50 µg/kg). Post-infarction mortality in this mouse model is about 30–40%, primarily occurring around days 4–7 after MI. Sham-operated mice (control) were subjected to the same protocol except that the artery was not ligated. The inhibitor was administered the 8 day after MI for the indicated period. Tissue were harvested after 12 weeks after MI. Left ventricle diastolic and systolic diameters were measured in anesthetized (isofluorane 1.5%) mice, according to the American Society of Echocardiography’s leading-edge method (using Vivid 7 echograph a 14 MHz probe). In addition, LV outflow velocity was measured by pulsed waves, and CO was calculated as follows:

${\text{CO}} = {\text{aortic VTI}} \times \left[ {\pi \times \left( {\text{LV outflow diameter/2}} \right)^{{2}} } \right] \times {\text{heart rate}},$ where VTI is velocity–time integral.

LV hemodynamic was assessed as described previously^11^. Briefly, mice were anesthetized (chloral 320 mg/kg, IP) and the carotid artery cannulated with a pressure–volume catheter (SPR839, Millar-Instruments, USA) and the catheter was advanced into the LV. Pressure–volume loops were obtained at baseline and during loading by gently occluding the abdominal aorta. LV end-systolic and end-diastolic pressures, dP/dt_max/min_, LV relaxation constant tau and were measured/calculated with IOX software (EMKA, France). Systolic BP was measured by tail-cuff plethysmography in trained conscious mice at weeks 8–10 using a BP2000 Visitech model. BP was measured every day in the same room at the same hour for 5 consecutive days. The BP measurements (expressed as mmHg) presented are the averages of the last 3 days.

Chronic kidney disease (CKD) was induced by subtotal nephrectomy in 8-weeks-old male mice (25–26 g). All surgeries were performed under ketamine/xylazine anaesthesia. Briefly, the left kidney was exposed, and the upper and lower poles were tied with a poly-glycolic acid suture line. The peritoneum and skin were then sutured, and the animals were returned to their individual cages. After 1 week of recovery, the second kidney was removed. Removal of the second kidney represents T0. Sham mice were subjected to the same surgical procedures but neither renal poles nor the right kidney were removed. Mice were monitored for any sign of distress, and those observed to be experiencing severe, unrelievable pain were euthanized. Renal failure was assessed by the measure of plasma creatinine and urea with an automatic analyser (Konelab 20i; Thermo Fisher Scientific, Vernon Hills, IL) at weeks 4 and 8 post-Nx.

### Histology and molecular biology

In the MI model, heart was harvested after hemodynamics and the atria and the ventricles were separated and weighed individually. A section of the left ventricle was immersed in Bouin fixative solution. In the CKD model, kidneys were harvested and a section was immersed in paraformaldehyde fixative solution. After fixation, the sections were dehydrated and embedded in paraffin. From these sections, 5-μm thick histologic slices were obtained and were stained with Sirius Red. For the measurement of cardiac or renal collagen density, slides were examined and photographed under a light microscope (Zeiss) at 40× magnification. Collagen content was calculated as percentage of collagen area to total area of the image. Perivascular collagen was excluded from the analysis. For tubular lesions scoring, kidney slides were stained with Periodic Acid-Schiff kit (Sigma) and the score of tubules with injury (epithelial cell necrosis and detachment, and tubular dilation) was analyzed blinded to the experimental groups.

#### Western-blot analysis

Total protein aliquots of 20 μg were prepared from cardiac homogenates and HCF and electrophoresed on SDS polyacrylamide gels and transferred to Hybond-c Extra nitrocellulose membranes (Amersham Biosciences). Membranes were incubated with primary antibodies for: Collagen I (Santa Cruz; dilution 1:500), Collagen type III (Santa Cruz; dilution 1:500), fibronectin (Millipore, 1:500), Galectin-3 (Thermo, 1:1000), α-SMA (Sigma, 1:1000), CTGF (Santa Cruz, 1:200), CD68 (Abcam, 1:500), CD80 (Santa Cruz, 1:500), CD86 (Santa Cruz, 1:500). Stain free detection was used for loading control (Bi-Rad Laboratories, CA, USA) After washing, the blots were incubated with peroxidase-conjugated secondary antibody, and binding revealed by ECL chemiluminescence (Amersham). After densitometric analyses, optical density values were expressed as arbitrary units. Results are expressed as an n-fold increase over the values of the control group in densitometric arbitrary units.

#### ELISA

IL6 and collagen type I concentrations were measured in cardiac tissue and cell supernatants by ELISA according to the manufacturer's instructions (R&D Systems).

#### Proliferation

Cell proliferation was assessed using the MTT Proliferation Assay (Sigma).

#### Real-time reverse transcription PCR

Frozen tissues (kidneys, heart) were homogenized in TRIzol (Life Technologies, Illinois, USA) using FastPrep beads (MP-Bio, CA, USA). cDNAs were generated using the Superscript II reverse transcriptase kit (Invitrogen,NY, USA), and qPCR was performed as previously described. Briefly, transcript levels were analysed in a CFX396 apparatus (Biorad). The reactions were performed in duplicate for each sample using the IQ SYBR Green supermix Kit (Biorad,CA, USA). To normalize gene expression, the geometric mean of multiple internal reference genes were used (*RS16*, *Ubc*, *Hprt* and *Gapdh* for mice experiments). Values in control conditions were set as 1 for each gene. The sequences of the specific primers are detailed in Table S8.

### Statistical analyses

All data are presented as the mean ± SEM. *p* < 0.05 was considered significant. Distributions were verified using the Kolmogorov–Smirnov test. Differences in the means between two groups for non-repeated variables were compared by Student’s *t* test. Differences in the means among groups and treatments were compared by one-way ANOVA for multiple comparisons, followed by Tukey’s correction for multiple comparisons (GraphPad).

## Supplementary Information

Supplementary Information.
