# Supplementary material for: Antifibrotic effect of novel neutrophil gelatinase-associated lipocalin inhibitors in cardiac and renal disease models
Source: Sci Rep. 2021 Jan 28;11:2591. doi: 10.1038/s41598-021-82279-0 (PMC7844219; doi:10.1038/s41598-021-82279-0)
Supplement: Supplementary file 1 — Supplementary Information. [file 41598_2021_82279_MOESM1_ESM.pdf]

ONLINE SUPPLEMENT

ANTIFIBROTIC EFFECT OF NOVEL NEUTROPHIL GELATINASE-ASSOCIATED LIPOCALIN INHIBITORS IN CARDIAC AND RENAL DISEASE MODELS

Benjamin Bonnard[1#], Ernesto Martínez-Martínez[1#], Amaya Fernández-Celis[2], Marie Pieronne-Deperrois[3], Quoc-Tuan Do[4], Isbaal Ramos[5], Patrick Rossignol[6], Faiez Zannad[6], Paul Mulder[3], Antoine Ouvrard-Pascaud[3], Natalia López-Andrés[2, 6*], Frédéric Jaisser[1, 6*]

[1]INSERM, UMRS 1138, Centre de Recherche des Cordeliers, Sorbonne Université, Université de Paris, Paris, France. [2]Cardiovascular Translational Research. Navarrabiomed (Miguel Servet Foundation), Instituto de Investigación Sanitaria de Navarra (IdiSNA), Pamplona. Spain. [3]Inserm U1096, UFR Médecine-Pharmacie, Rouen, France.[4]Greenpharma SAS, Orléans, France. [5]Innovative Technologies in Biological Systems SL (INNOPROT), Bizkaia, Spain. [6]Université de Lorraine, INSERM Centre d'Investigations Cliniques-Plurithématique 1433, UMR 1116, CHRU de Nancy, French-Clinical Research Infrastructure Network (F-CRIN) INI-CRCT, Nancy, France.

# These authors contributed equally to this work
* Both authors contributed equally to this study.
Corresponding authors:
Dr. Frédéric Jaisser
INSERM U1138, Centre de Recherche de Cordeliers, 15 rue de l'Ecole de Médecine, 75006, Paris, France. Tel: +33144278106. Fax: +33144276421
E-mail: frederic.jaisser@inserm.fr
Dr. Natalia López-Andrés.
Cardiovascular Translational Research. Navarrabiomed (Miguel Servet Foundation). C/Irunlarrea 3, 31008 Pamplona, Spain. Telephone: 34-848422359. Fax: 34-848422300
E-mail: natalia.lopez.andres@navarra.es

This file includes:

10 supplemental figure
8 supplemental tables

SUPPLEMENTAL FIGURES

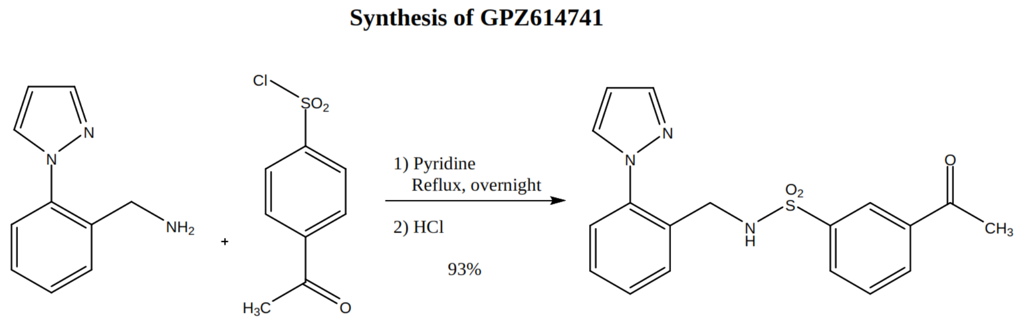

Figure S1: GPZ614741 synthesis scheme.

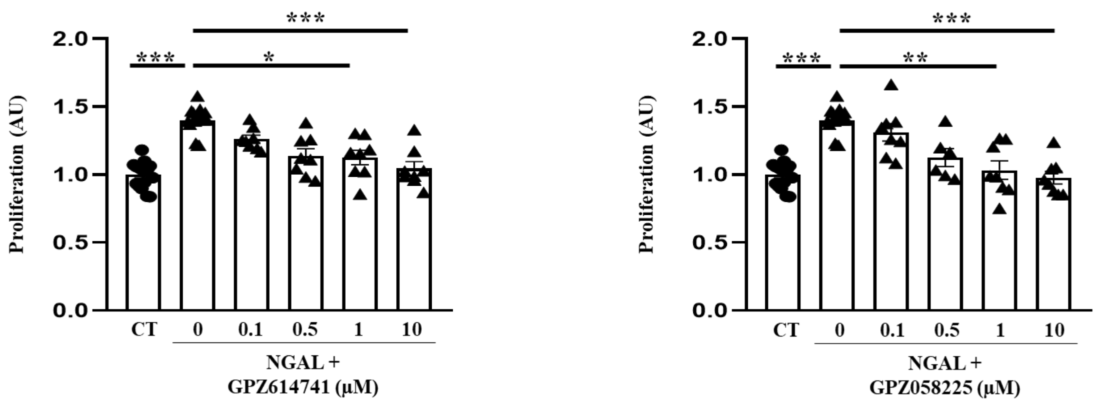

Figure S2: Dose-response of GP1 (GPZ614741) and GP2 (GPZ058225) on cell proliferation in hNGAL-treated human cardiac fibroblasts. * p< 0.05; ** p< 0.01 and *** p< 0.001.

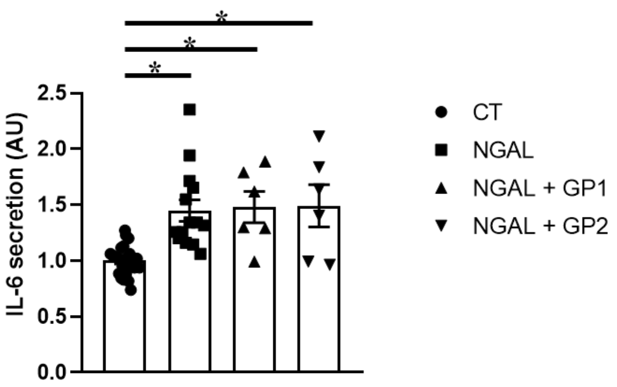

Figure S3: Effects of GP1 (GPZ614741) and GP2 (GPZ058225), at 1 µM on IL6 secretion in hNGAL-treated human cardiac fibroblasts. *p < 0.05

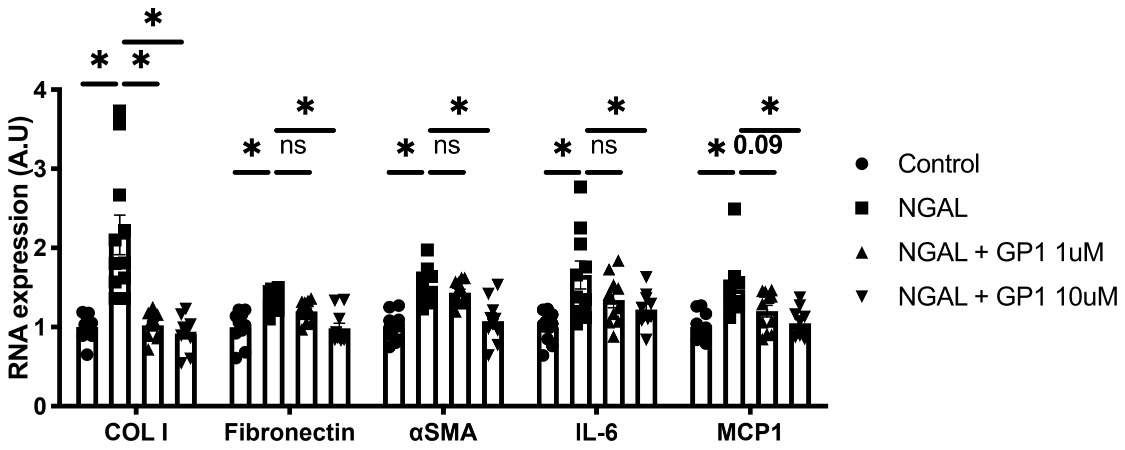

Figure S4: Effects of GP1 (GPZ614741) and GP2 (GPZ058225), at 1 μM and 10 μM on gene expression in mNGAL-treated mouse kidney fibroblasts. *p < 0.05

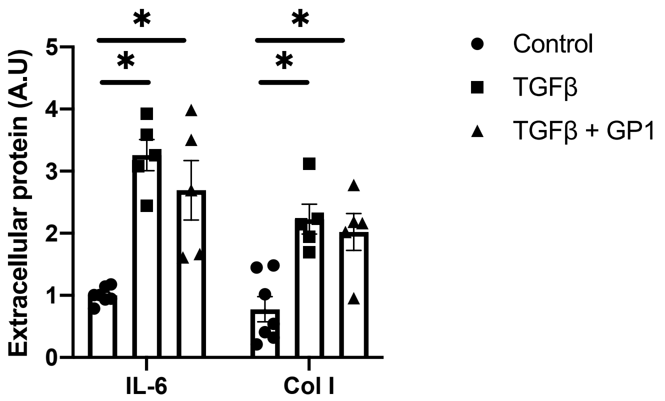

Figure S5: Effects of GP1 at 10 μM on IL-6 and Col I secretion in TGFβ-treated human cardiac fibroblasts. *p < 0.05

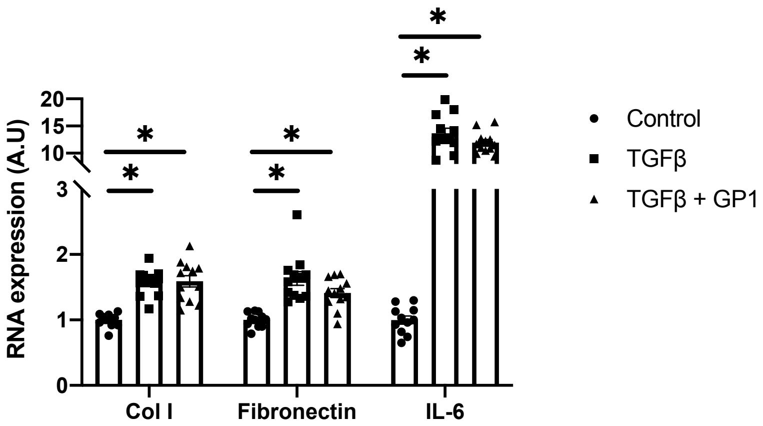

Figure S6: Effects of GP1 at 10 μM on gene expression in TGFβ -treated mouse kidney fibroblasts. *p < 0.05

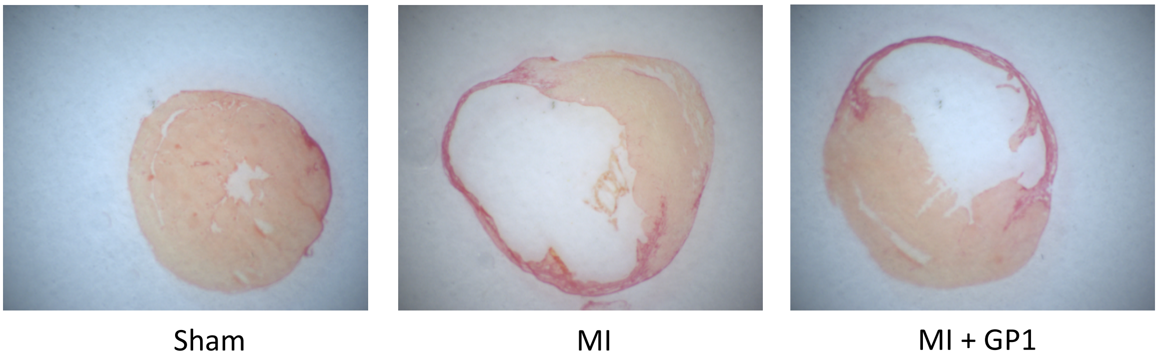

Figure S7: Representative picture of collagen deposition in infarct zone colored by sirius red staining.

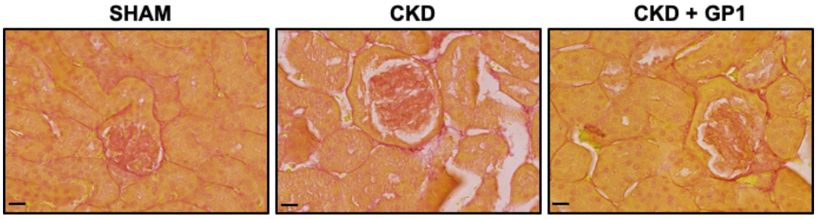

Figure S8: Glomerular fibrosis on kidney slide colored by sirius red staining.

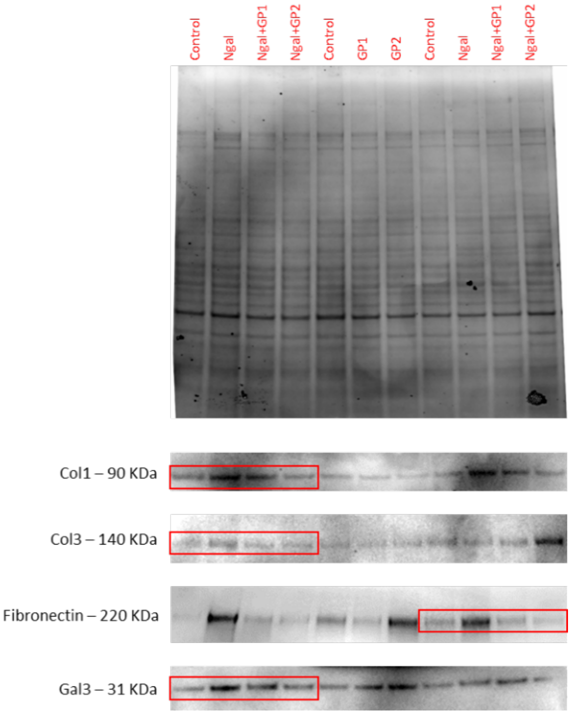

Figure S9: Original immunoblots and stain free gels for figure 1D;

C

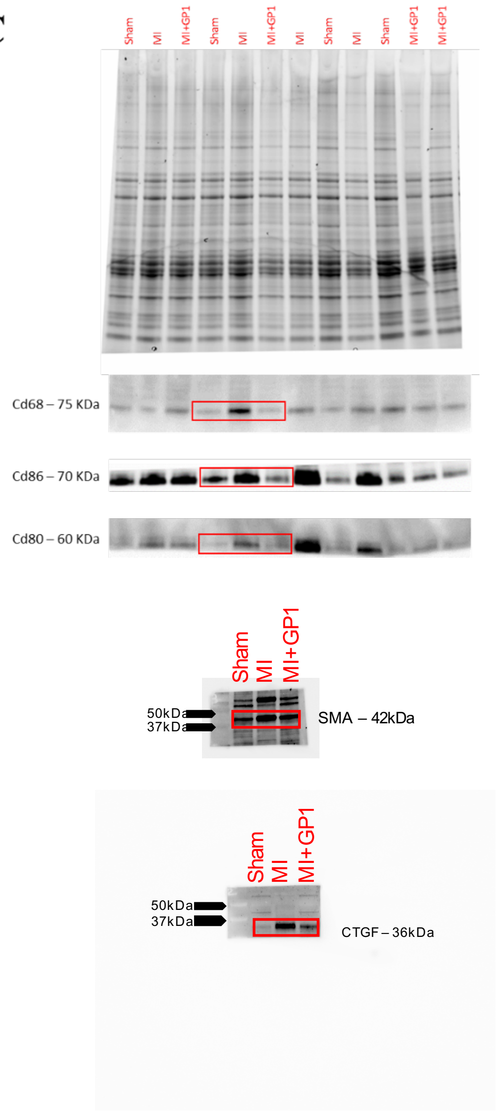

Figure S10: Original immunoblots and stain free gels for **(A)** figure 2C; **(B)** Figure 2D and **(C)** Figure 2E.

SUPPLEMENTAL TABLES

Table S1. Percentage of inhibition versus hNGAL on IL-6 secretion in human cardiac fibroblasts.

Compound	IL-6 secretion	% inhibition
Control	1.0 ± 0.1	
NGAL	2.21 ± 0.2 ***	
GPZ614741	0.86 ± 0.2 †††	61.1
GPZ058225	0.99 ± 0.3 ††	55.2
GPZ425915	1.12 ± 0.04 †	49.3
GPZ503502	1.32 ± 0.1 †	40.2
GPZ413473	1.35 ± 0.01 †	38.9
GPZ672150	1.55 ± 0.2	29.9
GPZ880710	1.57 ± 0.2	28.9
GPZ097126	1.58 ± 0.02	28.5
GPZ011720	1.60 ± 1.2	27.6
GPZ597101	1.61 ± 0.1	27.1
GPZ763971	1.69 ± 0.4	23.5
GPZ438190	1.75 ± 0.2	20.8
GPZ902633	1.81 ± 0.9	18.1
GPZ073952	1.82 ± 0.3 *	17.6
GPZ472686	1.98 ± 0.1 **	10.4
GPZ129400	1.99 ± 0.1 **	10.0
GPZ293019	2.01 ± 0.5 **	9.0
GPZ972604	2.02 ± 0.1 **	8.6
GPZ442790	2.04 ± 0.4 **	7.7
GPZ384799	2.05 ± 0.3 **	7.2
GPZ09424	2.16 ± 0.2 ***	2.3
GPZ062639	2.26 ± 0.3 ***	-2.3
GPZ982826	2.83 ± 0.2 ***	-28.0
GPZ469183	2.87 ± 0.2 ***	-29.9
GPZ069322	2.97 ± 0.5 ***	-34.4
GPZ192583	3.19 ± 0.3 ***†	-44.3
GPZ128506	5.72 ± 3.5***†††	-158.8
GPZ574458	6.28 ± 1.6***†††	-184.2
GPZ422811	8.87 ± 0.4***†††	-301.3
GPZ291090	Poor solubility	
GPZ347499	Toxic	
GPZ048130	Toxic	

Values are mean ± SEM. *p<0.05; **p<0.01; ***p<0.001 vs. Control. †p<0.05; ††p<0.01; †††p<0.001 vs. NGAL-treated human cardiac fibroblasts.

Table S2. Percentage of inhibition versus hNGAL on Col I, Col III, Fibronectin and Galectin 3 protein levels in human cardiac fibroblasts.

**Compound**	**Col I synthesis**	**% inhibition**
Control	1.0 ± 0.3	
NGAL	2.03 ± 0.3 *	
GPZ614741	1.09 ± 0.2 †	46.4
GPZ058225	1.27 ± 0.1 †	37.5
GPZ425915	1.27 ± 0.3 †	37.5
GPZ413473	0.99 ± 0.3 †	51.2
**Compound**	**Col III synthesis**	**% inhibition**
Control	1.0 ± 0.2	
NGAL	1.67 ± 0.2 *	
GPZ614741	1.05 ± 0.1	37.1
GPZ058225	1.05 ± 0.1	37.1
GPZ425915	1.41 ± 0.5	15.6
GPZ413473	1.96 ± 0.3 *	-17.4

**Compound**	**Fibronectin synthesis**	**% inhibition**
Control	1.0 ± 0.2	
NGAL	1.8 ± 0.3 *	
GPZ614741	0.95 ± 0.2 †	47.2
GPZ058225	0.62 ± 0.1†	65.5
GPZ425915	1.1 ± 0.2	38.9
GPZ413473	1.6 ± 0.3	11.1
**Compound**	**Galectin-3 synthesis**	**% inhibition**
Control	1.0 ± 0.2	
NGAL	2.1 ± 0.3 *	
GPZ614741	1.3 ± 0.1	38.1
GPZ058225	1.0 ± 0.2†	52.3
GPZ425915	1.3 ± 0.1	38.1
GPZ413473	1.4 ± 0.1	33.3

Values are mean ± SEM. *p<0.05; **p<0.01; ***p<0.001 vs. Control. †p<0.05; ††p<0.01; †††p<0.001 vs. NGAL-treated human cardiac fibroblasts.

Table S3. Chemical formula of GPZ614741 (CAS: 1241512-52-6) **GP1** and GPZ058225 (CAS: 519050-14-7) **GP2.**

Compound name	Corresponding chemical formula
GPZ614741 (CAS: 1241512-52-6) **GP1**	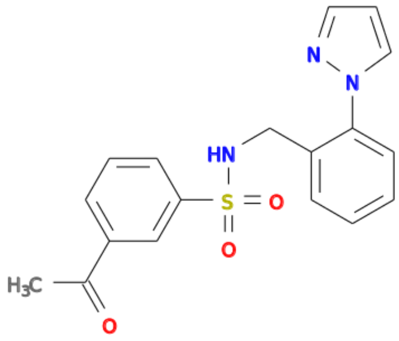
GPZ058225 (CAS: 519050-14-7) **GP2**	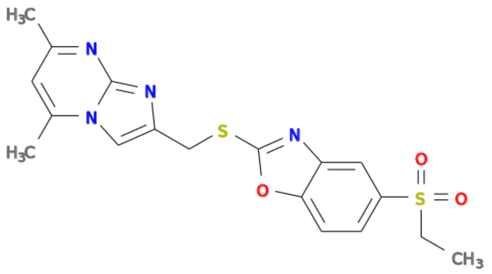

Table S4. Second generation compounds derived from GP1 and GP2. Percentage of inhibition versus hNGAL on IL-6 secretion in human cardiac fibroblasts.

Original compound	Compound	IL-6 secretion	% inhibition
	Control	1.00 ± 0.03	
	NGAL	1.45 ± 0.1**	
GPZ614741		0.59 ± 0.02††	59.5
GPZ614741	GPZ642292	0.64± 0.02††	55.9
GPZ614741	GPZ478519	0.69± 0.02††	52.4
GPZ614741	GPZ505884	0.83± 0.02††	42.8
GPZ614741	GPZ778195	0.88± 0.04††	39.3
GPZ614741	GPZ595600	0.92± 0.04††	36.6
GPZ614741	GPZ863205	0.93± 0.03††	35.9
GPZ614741	GPZ913629	0.93± 0.02††	35.9
GPZ614741	GPZ200962	1.18 ± 0.2†	18.6
GPZ614741	GPZ385963	1.31 ± 0.1	9.7
GPZ614741	GPZ372533	1.40 ± 0.2*	3.4
GPZ614741	GPZ524806	1.46 ± 0.4	-0.7
GPZ058225		0.72 ± 0.02††	50.4
GPZ058225	GPZ564849	0.85± 0.1††	41.4
GPZ058225	GPZ278618	0.86± 0.02††	40.1
GPZ058225	GPZ743042	0.94 ± 0.03††	35.2
GPZ058225	GPZ519431	1.02± 0.1††	29.7
GPZ058225	GPZ469120	1.20 ± 0.1†	17.2
GPZ058225	GPZ406487	1.21 ± 0.1†	16.5
GPZ058225	GPZ827496	1.22 ± 0.02†	15.9
GPZ058225	GPZ380975	1.53 ± 0.3*	-5.5
GPZ058225	GPZ925853	1.63 ± 0.4*	-12.4
GPZ058225	GPZ998905	1.67 ± 0.1**	-15.2
GPZ058225	GPZ228729	1.68 ± 0.3**	-15.9
GPZ058225	GPZ241243	Toxic	

Values are mean ± SEM. *p<0.05; **p<0.01; ***p<0.001 vs. Control. †p<0.05; ††p<0.01; †††p<0.001 vs. NGAL-treated human cardiac fibroblasts.

Table S5. ***Cell toxicity and ADME.*** 1) Hepato- and cardio- toxicity determination; 2) ADME drug incompatibility test (CYP3A4 inhibition potential); 3) ADME parameters determination.

**Compounds**	**TOXICITY**		**ADME**			
	Hepatotoxicity μM	Cardiotoxicity % (100= no toxicity)	Aquous solubility %	Chemical Stability %	hPPB%	CyP 3A4 Inhibition % (100= no inhibition)
**GP7058225**	No tox up 100 uM	101.73 ±4.1	63.46	92.2	89.11	68.07±1.52
**GPZ614741**	No tox up 100 uM	101.76±2.48	100	103	71.16	72.21±1.94

Table S6: Ambinter code for first generation compounds.

Compound	Ambinter Code
GPZ614741	Amb11184725
GPZ058225	Amb2248638
GPZ425915	Amb3442133
GPZ503502	Amb11202635
GPZ413473	Amb3489508
GPZ672150	Amb4231111
GPZ880710	Amb2050586
GPZ097126	Amb278968
GPZ011720	Amb10845008
GPZ597101	Amb8628398
GPZ763971	Amb10758566
GPZ438190	Amb535844
GPZ902633	Amb28974544
GPZ073952	Amb2412316
GPZ472686	Amb10877513
GPZ129400	Amb13907749
GPZ293019	Amb20418710
GPZ972604	Amb7039537
GPZ442790	Amb10663816
GPZ384799	Amb24107065
GPZ09424	Amb10841302
GPZ062639	Amb10200435
GPZ982826	Amb9982824
GPZ469183	Amb6889815
GPZ069322	Amb1043689
GPZ192583	Amb21856038
GPZ128506	Amb10810342
GPZ574458	Amb18511830
GPZ422811	Amb28974546
GPZ291090	Amb7809477
GPZ347499	Amb9154868
GPZ048130	Amb21856064

Table S7: Ambinter code for second generation compounds.

Compound	Ambinter Code
GPZ478519	Amb9191218
GPZ505884	Amb8210069
GPZ595600	Amb14471392
GPZ642292	Amb8245375
GPZ778195	Amb19510760
GPZ863205	Amb22723246
GPZ913629	Amb14013470
GPZ278618	Amb20642011
GPZ519431	Amb1347727
GPZ564849	Amb2602481
GPZ743042	Amb1262669
GPZ200962	Amb11195808
GPZ469120	Amb2250238
GPZ406487	Amb1346372
GPZ827496	Amb2250270
GPZ385963	Amb11950437
GPZ372533	Amb10618628
GPZ524806	Amb14019491
GPZ380975	Amb2250231
GPZ925853	Amb13134
GPZ998905	Amb1148393
GPZ228729	Amb1145445
GPZ241243	Amb1148474

Table S8: Primers used in mice in real time PCR analysis

	PRIMER	FOWARD	REVERSE
**Housekeeping genes**	*HPRT*	TCTAACTTTAACTGGAAAGAATGTC	TCCTTTTCACCAGCAAGCT
	UBC	CGGAGTCGCCCGAGGTCACA	GGGCTCGACCTCCAGGGTGAT
**Target genes**	*αSMA*	TGTGCTGGACTCTGGAGATG	GAAGGAATAGCCACGCTCAG
	CD68	ACAAGGGACACTTCGGGCCA	GTCGTCTGCGGGTGATGCAG
	CD80	TTCACCTGGGAAAAACCCCCAGAA	ACGACGACTGTTATTACTGCGCC
	CD86	AGCAGACGCGTAAGAGTGGCT	CATGGTGCATCTGGGGTCCATC
	Collagen I	CCCCGGGACTCCTGGACTT	GCTCCGACACGCCCTCTCTC
	Fibronectin	CCT ACG GCC ACT GTG TCA CC	AGT CTG GGT CAC GGC TGT CT
	IL6	CTCTGGGAAATCGTGGAAATG	AAGTGCATCATCGTTGTTCATACA
	MCP1	GGCTGGAGAGCTACAAGAGG	TCTTGAGCTTGGTGACAAAAAC
	TNFα	GCCTCTTCTCATTCCTGCTTG	CTGATGAGAGGGAGGCCATT

Reply:

For the wholes images, unfortunately we are not able to provide them. For the western blot studies, we used criterion TGX gels already prepared from Bio-Rad Company. These gels give us several advantages such as the percentage of polyacrylamide which is in gradient concentration (between 4-15%) and that allow us the separation in the electrophoresis of proteins from low to high molecular weight. After that, we transfer the proteins into the membranes and we verify that proteins have been transferred accurately.

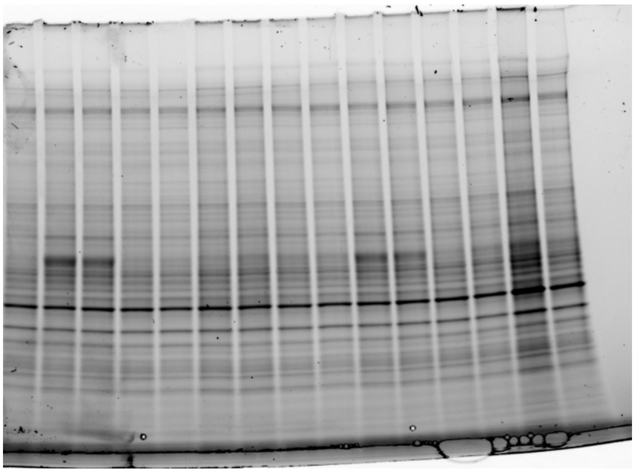

Figure 1. Whole gel (Stain free technology).

We cannot include full-length blots because once we transfer the proteins into the membranes; we cut the membranes into separate pieces which include the selected protein. Firstly, we optimize the antibody concentration probing that it detects only one band. Each piece of membrane is incubated with the primary antibody of interest and an HRP-conjugated secondary antibody. Afterwards, we add a chemiluminescent substrate to the blot. Then, we use the chemiluminiscence imaging equipment (Bio-Rad). The system will quickly assess the blot and then capture an image automatically that allows us an extremely accurate quantification. Once the dilution of the primary antibody and its specificity has been probed, we cut the membranes (with the samples of interest) in two pieces in order to optimize the resources and to reduce the quantity of antibodies. Finally,

we use a stripping solution to re-incubate the membranes with another antibody. We show an example for your consideration.

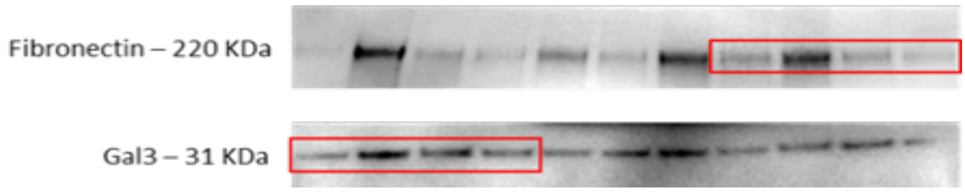

Figure 2. In the same western blot we performed fibronectin and Galectin-3 immunodetections, because the molecular weights are very different and the bands are clearly separated.

About the Figure 3C, the original image presented in manuscript is from the uncropped picture (Figure 3). The difference between the original image in manuscript and the picture showed in supplementary information is due to brightness/contrast modification.

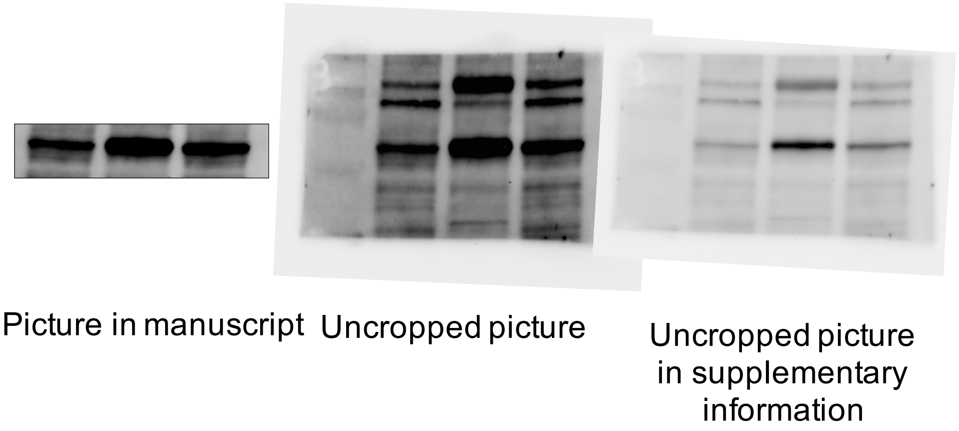

Figure 3. Image process
